# Establishment of a Robot-Assisted Bronchoscopy Program at a Veterans Affairs Hospital

**DOI:** 10.7759/cureus.74013

**Published:** 2024-11-19

**Authors:** Bianka Eperjesiova, Jessica Peterson, P S Sriram

**Affiliations:** 1 Pulmonology, Critical Care, Sleep Medicine, and Immunology, Malcom Randall Veteran Affairs Medical Center, Gainesville, USA; 2 North Florida Foundation of Research and Education, Malcom Randall Veteran Affairs Medical Center, Gainesville, USA; 3 Kinesiology, New Mexico State University, Las Cruces, USA; 4 Pulmonology, Critical Care, and Sleep Medicine, University of Florida College of Medicine, Gainesville, USA

**Keywords:** lung cancer detection, lung nodule, peripheral navigation bronchoscopy, program development, robot-assisted bronchoscopy, veterans health

## Abstract

Lung cancer has high mortality rates attributed to late diagnosis and treatment. Robot-assisted bronchoscopy (RAB) offers promising solutions to these challenges, enabling precise navigation and biopsy of small and difficult-to-reach lung nodules. We present the early outcomes and challenges encountered in establishing an RAB program at a Veterans Affairs (VA) Medical Center. The experience of establishing infrastructure, the allocation of resources, and forming a multidisciplinary team is discussed. Challenges such as funding allocation, technological integration, and logistical hurdles are addressed. Despite challenges, the successful establishment of an RAB program demonstrates the transformative potential in enhancing diagnostic precision and patient outcomes within a VA hospital, thus providing a blueprint for nationwide adoption. Future directions include expanding procedural capacity through workforce training, leveraging advanced imaging technology, and establishing a training center for other VA RAB teams. The integration of RAB into VA healthcare systems represents advancements in lung cancer care for veterans.

## Introduction

In 2023, the United States detected upward of 238,000 new cases of lung cancer, and remains the leading cause of cancer-related deaths, with 120-160,000 deaths annually [1]. In addition to common risk factors like age and smoking, veterans experience heightened lung cancer risks due to service-related exposures and are 25% more likely to receive a lung cancer diagnosis compared to non-veterans [2]. This highlights the challenge of detecting lung cancer early, as symptoms often emerge late in its progression, delaying diagnosis and treatment. Additionally, the peripheral and difficult-to-reach location of the nodules complicates early detection through inconclusive physical examination, while screening computed tomography (CT) scans can miss small nodules or yield false positives [3].

Robot-assisted bronchoscopy (RAB) has been a promising solution to these challenges in peripheral sampling. FDA-approved robotic platforms offer advanced capabilities for navigating and sampling difficult-to-reach lung nodules, marking advancements in lung cancer diagnostics [4]. Currently, the Veterans Affairs (VA) owns six Johnson and Johnson electromagnetic navigation Monarch (Johnson and Johnson MedTech, New Jersey, United States) and 47 Intuitive shape-sensing Ion (Intuitive Surgical, Inc., California, United States) RAB platforms. The VA uses Ion systems due to their reduced reliance on disposables, making them a more cost-effective and environmentally friendly choice. RAB enhances bronchoscopic procedures by improving diagnostic yield, precision, maneuverability, and visualization, reducing operator fatigue, ensuring safety, and expanding capabilities for future therapeutics [4-6]. Each RAB is performed in conjunction with linear endobronchial ultrasound (EBUS) for mediastinal staging. 

While RAB shows promise with improved diagnostic yield and health outcomes compared to other methods, the integration of RAB into patient care plans presents challenges, particularly within VA hospitals [6,7]. We illustrate our journey in establishing a veteran-focused multidisciplinary RAB program. Through our experience, we aim to provide the challenges and successes of an RAB program implementation with additional information and context about VA healthcare settings.

## Technical report

Producing data in support of RAB at our facility

The North Florida South Georgia Veterans Health Administration (NF/SG VHA) network spans 26 locations across 50 counties, encompassing 40,000 square miles, making it the largest in the nation. In 2021, 176,915 veterans received healthcare within NF/SG VHA. According to Health Factor Data within the Computerized Patient Records System (CPRS), around 30,000 of those veterans are eligible for lung cancer screening.

**Figure 1:**
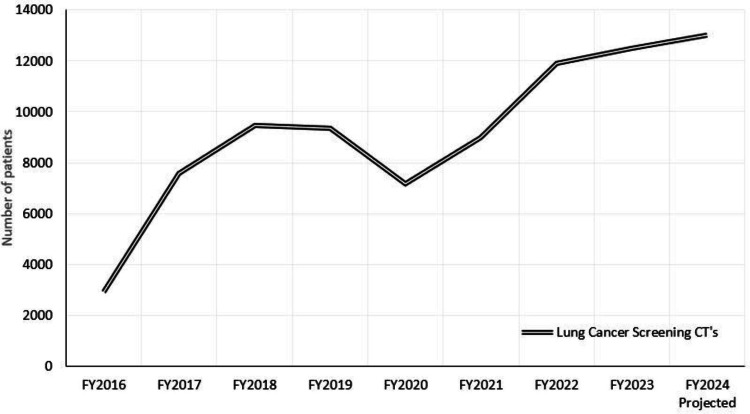
Total number of lung cancer screening CTs performed per fiscal year (FY). Image credits: Dr. Peterson created the figure in Excel using the graph function from data that was collected by Dr. Eperjesiova

The lung cancer screening program is led by a pulmonologist and an advanced registered nurse practitioner (ARNP). The data is managed by a dedicated team of eight lung cancer screening nursing coordinators in the National Center of Lung Cancer Screening platform. A preliminary examination of radiology services for lung cancer screening via CT chest examinations was conducted for fiscal years (FY) 2016-2020 (pre-RAB) to project future RAB caseload and referral patterns at the referral center, Malcom Randall Veterans Affairs Medical Center (VAMC). Figure 1 represents the total lung cancer screening CTs performed across our VA network during the specified fiscal years.

There are two additional referral pathways for lung cancer detection: incidental lung nodules and nodules detected during cancer surveillance. Incidental lung nodules are discovered on imaging not specifically intended for lung cancer screening and are assessed by Fleischner criteria [8] with a radiology recommendation for a virtual consultation. Nodules detected during cancer surveillance are typically seen within immediate post-treatment years and are generally identified during serial radiologic follow-up.

**Figure 2:**
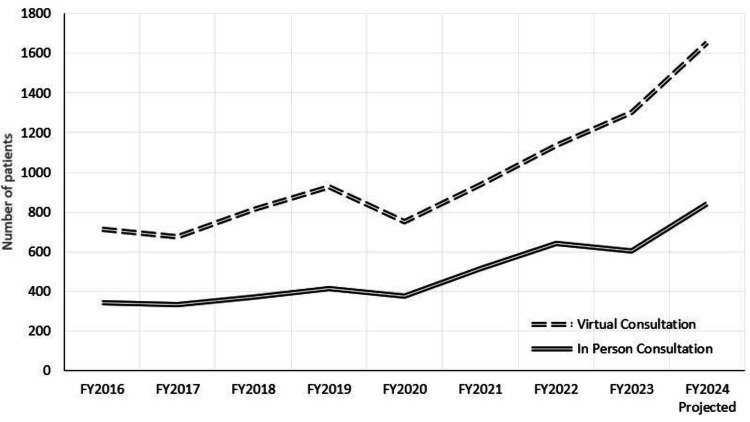
Total number of virtual consultations (dashed lines) and in-person consultations (solid lines) per fiscal year (FY). Image Credits: Dr. Peterson created the figure using the graph function in Excel using data collected by Dr. Eperjesiova

From FY2016 to Q2 FY2024, the number of virtual consultations has shown an upward trend (Figure 2). The dip in FY2020 likely occurred due to COVID-related priorities, which led to the suspension of routine screenings and surveillance, as well as patient hesitancy. Similarly, while the trend was steady in 2016-2021, there has been a consistent increase in the number of in-person consultations since launching the RAB program. Approximately 60% of our in-person consultations are requested for abnormal screening CTs, while the remaining 40% are for abnormal incidental findings and lung cancer surveillance imaging.

**Figure 3:**
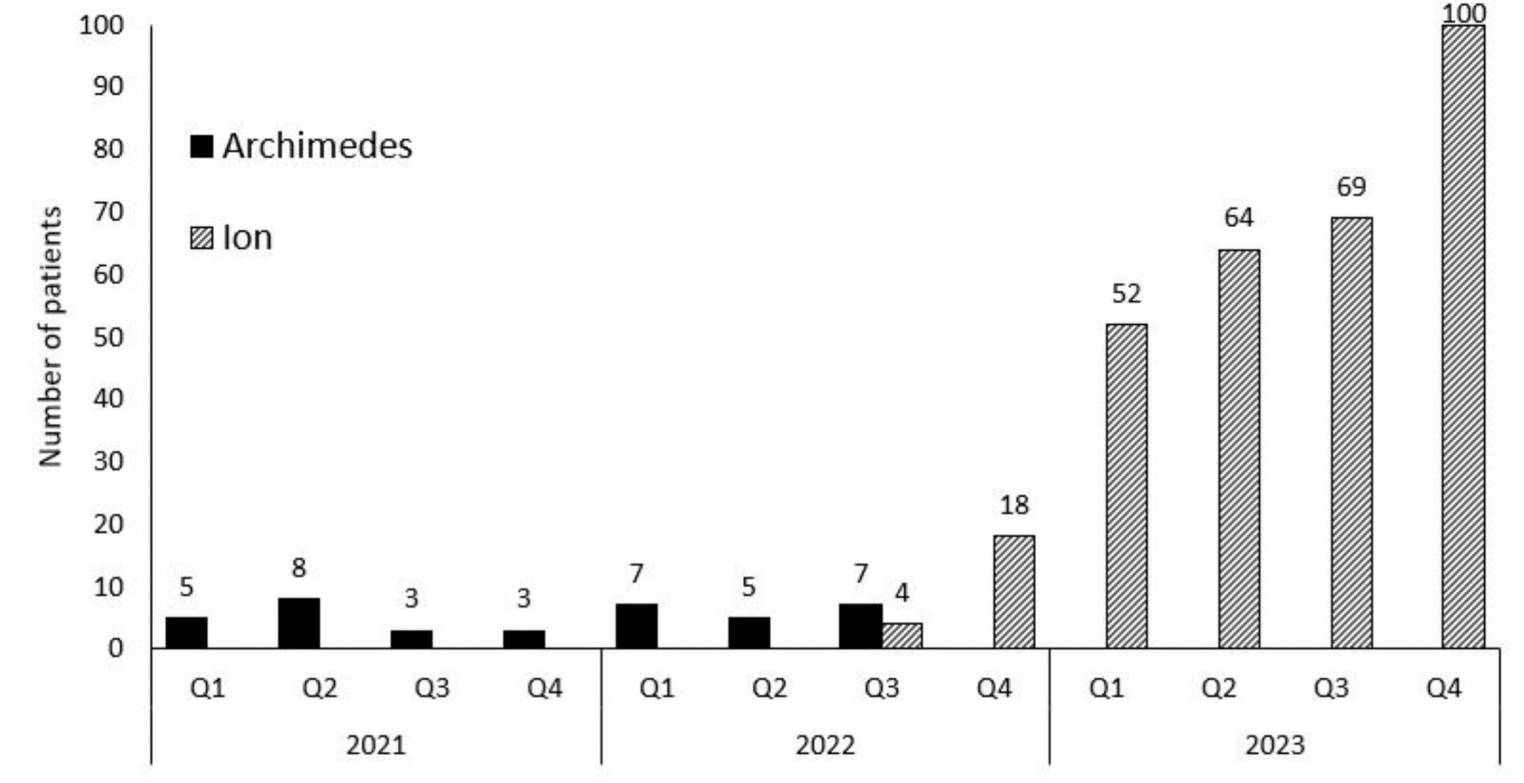
Total number of bronchoscopic navigation procedures split by quarter showing the use of Archimedes platform (block color) and Ion RAB platform (lined). Image credits: Dr. Peterson created the figure using the graph function in Excel with the data collected by Dr. Eperjesiova

Previous diagnostic technology yielded a low total number of procedures when comparing the quarterly trend of the previous approach (non-robotic peripheral navigation, diagnostic rate below 70%) with the current methodology (Figure 3). Since the launch of RAB, the number of diagnostic procedures conducted has increased by approximately 850%. 

Obtaining and allocating funds

The acquisition of RAB at the Malcom Randall VAMC marked a departure from traditional funding mechanisms such as the Lung Precision Oncology Program, Veterans Integrated Service Networks (VISN), or national allocation, as it was funded locally. The Strategic Equipment Planning Guide committee approved the Electronic Equipment Request (SEPG-EER) after recognizing Ion's significance as a strategic, innovative, high-priority investment and a proactive response to elevate the standard of care for veterans. 

Putting together a multidisciplinary diagnostic thoracic oncology team

Launching a multidisciplinary RAB program necessitates establishing the required infrastructure and providing adequate training. The team includes an interventional pulmonologist and an advanced bronchoscopist who review virtual consultations, offer recommendations for the next steps, and perform the procedures. Two advanced practice providers (APP) evaluate patients for physician-recommended diagnostic or therapeutic procedures, coordinate the logistics of bronchoscopic procedures, share results with patients, and coordinate treatment. Respiratory therapists (RT), anesthesiologists and certified registered nurse anesthetists (CRNAs), endoscopy nursing staff, cytologists, pathologists, and rotating sterile processing department (SPD) staff complete the core team.

Partnerships with various supporting services have also been established. Multidisciplinary tumor board members and other pulmonologists often initiate referrals to expedite diagnosis and treatment. Purchasing and prosthetics procure necessary disposables. Finally, biomedical engineering ensures technical support and maintenance of equipment. 

Infrastructure and resources

Bronchoscopy Suite

There are two bronchoscopy suites in the endoscopy outpatient area. Both suites have cutting-edge technology like the Intuitive Ion RAB platform (Model IF1000, Intuitive Surgical, Inc., California, United States), Olympus linear ultrasound bronchoscopes (Model BF-UC180F, Olympus America Inc., Pennsylvania, USA), radial probes (Model UM-S20-17S, Olympus America Inc., Pennsylvania, USA), and C-arm 2D fluoroscopy (Model 718131, Koninklijke Philips N.V., Amsterdam, Netherlands). The larger, 312-square-foot suite has additional tools (laser therapy, argon plasma coagulation, cryotherapy, metallic self-expanding, silicone stents, and other hemostatic devices). 

Radiology Services

Same-day CT chest protocol has been implemented for all RAB patients. Integrated imaging capabilities allow direct, prioritized transmission of CT images to our robotic computer system, reducing turnaround time. Our scheduling protocol optimizes procedural efficiency and minimizes disruptions. 

Anesthesia Services

General anesthesia is mandatory for feasibility and patient safety. Customized anesthesia protocol minimizes atelectasis and CT-to-body divergence (CTBD). A dedicated medication dispenser contains standard anesthesia medications but also epinephrine, tranexamic acid for hemostasis, and indocyanine green for nodule marking. Close collaboration between anesthesia and the proceduralist is critical. Anesthesia support is further enhanced by permanent anesthesia equipment in the procedure room, eliminating the need for daily equipment transport. 

Performing RAB/EBUS

The robotic computer system is used to plan multiple paths based on the same-day CT scan. After patient intubation, robotic technology is registered and navigated to the target. Next, assessment with a radial probe and fluoroscopy allows for accurate fine needle aspiration, forceps biopsy, a 1.1-mm cryoprobe, a cytology brush, and bronchoalveolar lavage sampling. A rapid onsite evaluation (ROSE) cytopathologist provides real-time feedback, ensuring adequate tissue acquisition, and even guiding further sampling. Once peripheral sampling is completed, linear EBUS sampling is performed by a senior pulmonary critical care fellow.

Assistant Staffing

Respiratory therapy (RT) staffing has adapted to operational needs by hiring two and up-training two RTs to assist with setup, procedural assistance, breakdown, and room turnover. While one RT is assigned to the suite, a second circulating RT is called after the RAB procedure to assist solely with robot turnover, ensuring streamlined operations for maximum productivity.

Sterile Processing

Acquiring high-level disinfection cleaning equipment during Ion acquisition was imperative, despite adding to the overall cost. Intuitive Ion's use of reusable accessories requires specialized expertise for effective reprocessing. In contrast, other robot platforms opt for disposable tools, circumventing the need for SPD involvement. The latter strategy introduces its own set of challenges, relying heavily on timely procurement to maintain optimal inventory levels, making it more susceptible to the persistent risk of supply chain disruptions. 

Standard of care plan from nodule discovery to treatment

**Figure 4:**
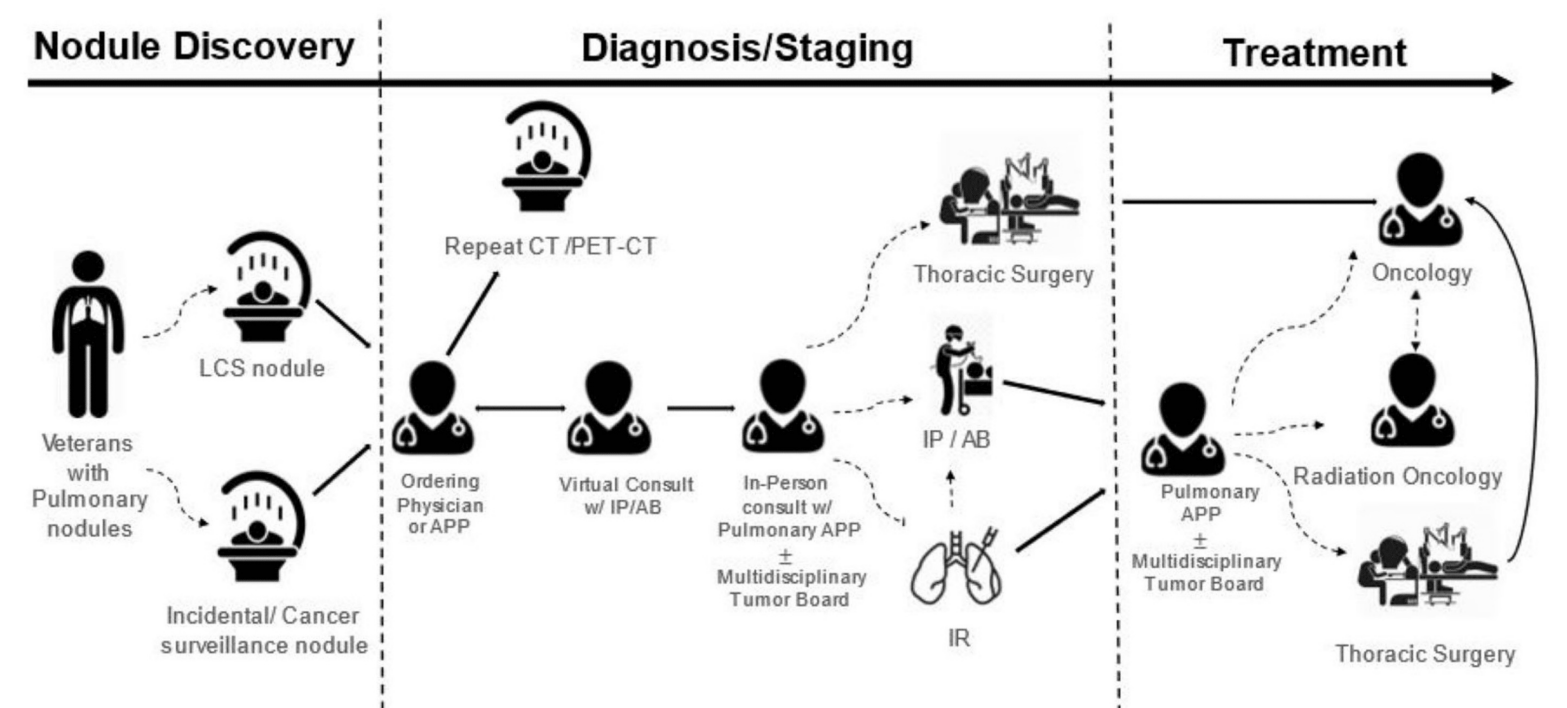
Systematic workflow of the multidisciplinary lung nodule program, beginning with identifying nodules in veterans and progressing through the various stages of treatment. Solid line arrows are mandatory steps in the workflow; dashed line arrows are optional. Upon discovery of a nodule, whether lung cancer screening, incidental, or through cancer surveillance, radiology advises subsequent actions according to evidence-based medicine criteria and further suggests to consult our lung nodule clinic. The physician, APP, or lung cancer screening nurse who ordered the CT scan would either order repeat chest CT at the suggested interval or initiate virtual consultation involving a fellow and a proceduralist to discuss findings. This consultation team delivers findings. If repeat chest CT or diagnostic PET-CT scans are necessary, they will be ordered by the referring physician or APP, and virtual consultation will be resubmitted once the results are available. If procedure is needed, the specific procedure (e.g., RAB/EBUS, EBUS alone, transthoracic needle aspiration, or thoracic resection) is recommended by the proceduralist during a virtual visit. The patient is then scheduled for an in-person consultation with the PA or ARNP, who conducts a comprehensive assessment, reviews the medical history, performs a physical examination, and reviews the pulmonary function test to evaluate the patient's suitability for the procedure. Additionally, the patient is educated about the procedure, associated risks and benefits, and pulmonary consents are obtained if indicated. Then, a procedure is performed. Once final pathology has been reported, the proceduralist recommends treatment options. The PA or ARNP engages in discussion regarding findings from the biopsy and next steps. Multidisciplinary tumor board is engaged if necessary, and patients are then referred for treatment. PA: physician assistant; ARNP: advanced registered nurse practitioner; LCS: lung cancer screening; EBUS: endobronchial ultrasound; RAB: robot-assisted bronchoscopy; APP: advanced practice provider Stock images from Word and Intuitive Surgical. Workflow designed by Dr. Eperjesiova.

The RAB program at Malcom Randall VAMC begins with the review of CT chest scans, identifying patients needing further assessment. This is followed by a diagnostic procedure and then the formulation of a treatment plan (Figure 4). 

This study was approved by both the University of Florida and NF/SG VA Institutional Review Board with the approval numbers; IRB202400034 and 1789412-2 respectively.

Results

Installation and Training 

Multiple training sessions with SPD prior to the official launch were conducted once the RAB equipment was delivered. Collaborative dry run sessions and meetings were held, culminating in the successful execution of the inaugural procedure.

The primary proceduralist for RAB/EBUS at Malcom Randall VAMC has completed a fellowship in interventional pulmonology, specializing in various airway and pleural procedures, including RAB/EBUS. The rest of the team has undergone specialized training through the Intuitive program in Atlanta.

To enhance our capabilities, we procured a second Intuitive Ion platform and launched our first simulation lab. This lab offers a training curriculum for fellows in RAB/EBUS procedures, 23 of whom have completed the program during the first four months of operation. The lab provides comprehensive education and hands-on experience in interventional pulmonary procedures, promoting technical proficiency and collaborative learning. We expect this to improve procedural competency and patient care. Additionally, nationwide VA teams visit our facility for structured case observations and hands-on lab simulations, advancing the skills and knowledge of VA RAB teams and improving care within the VA system.

Initial RAB cases

The data presented herein were obtained by extracting relevant information from the Malcom Randall VAMC electronic health records system. Simple descriptive statistics were performed in Microsoft Excel (version 2308, Microsoft Corporation, Redmond, Washington, United States) using means and standard deviations for continuous variables and N and percentages for nominal variables.

Nodule Discovered

In the Malcom Randall VAMC system, 8,992 screening CT chest scans were performed in FY2021, increasing to 11,902 in FY2022 and 12,315 in FY2023. The center is projected to perform about 15,000 scans in FY2024, showing a consistent upward trend. Approximately 900 veterans will have Lung Imaging Reporting and Data System (Lung-RADS) 4A or 4B findings in 2024, indicating lesions requiring biopsy or immediate treatment.

*Diagnosis/Stage* 

Our preliminary cohort of patients (N=338) that underwent RAB/EBUS were mostly male, with 314 (92%) participants compared to 24 females, spanning ages from 42 to 92 years, with an average age of 71.9 (±7.7). Among the patients, 171 (50.6%) were referred for in-person consultation at the lung mass clinic after abnormal lung cancer screening, while 163 (48.2%) patients were referred after the discovery of incidental nodules. The average length of the procedure when sampling one nodule was 76.12 minutes. However, in the first 10% of cases, the average procedure length was 88.12 minutes vs. the most recent 10% of cases, where the procedure length was 69.44 minutes, demonstrating a learning curve observed by a reduced procedural time of approximately 18 minutes. The size of the primary target, measured in millimeters, ranged from 5 mm to 86 mm, with an average size of 20.73 mm (±0.72). A total of 458 nodules were biopsied. Overall, we were able to detect 231 (68.3%) patients with malignancies, and 99 (29.3%) patients had non-malignant samples (i.e., sarcoidosis, interstitial lung disease, infection, and benign inflammation). When using strict diagnostic criteria determined originally by the NAVIGATE study, 303 (89.6%) patients received a diagnosis from the first RAB/EBUS procedure [9]. The twelve-month diagnostic yield was calculated per subject as the rate of true positives plus true negatives of all subjects with attempted lung lesion biopsies as provided by the NAVIGATE study. Furthermore, 10 patients (2.9%) required a repeat RAB/EBUS (due to an inconclusive first procedure), five patients (1.4%) required additional trans-thoracic needle aspiration, six patients (1.7%) were empirically radiated, and two patients (<1%) required surgical resection for diagnosis. The remaining 12 patients (3.4%) are awaiting diagnosis at the time of writing. 

Treatment

Of those that have received a diagnosis following RAB/EBUS procedure (326 patients), 26 (7.9%) underwent surgical resection, 132 (40.2%) received radiation, 101 (31.1%) received other oncological care, and 95 (29%) were treated by pulmonology for the infectious or inflammatory process. It is important to note that some of the patients are awaiting treatment at the time of writing and that some patients underwent multiple treatment types. 

## Discussion

Importance of lung cancer and early detection screening in the VA population

Veterans have a higher rate of lung cancer and a lower rate of survival than the general population [10]. Implementing screening and diagnostic programs for lung cancer within the VA healthcare system can significantly enhance survival rates by detecting the disease early when treatment is most effective [2]. Addressing challenges such as late symptom onset and ensuring access to screening services is imperative. Beyond conventional risk factors like age and smoking, veterans confront elevated lung cancer risks due to service-related exposures [11]. Unfortunately, the US Preventive Services Task Force (USPSTF) fails to account for these factors in screening criteria [12], potentially missing cases despite known or suspected carcinogenic effects. In FY 2024, approximately 15,000 screening CTs are projected across all NF/SG VHA network facilities, accounting for about half of eligible veterans. Based on data from previous fiscal years, about 6% of these screenings are projected to result in a lung-RADS score of 4A or 4B, necessitating either biopsy or immediate treatment interventions, thus allowing us to predict our potential workload with the RAB. From our experience, lung nodules in veterans are as likely to be detected incidentally as they are to be detected through age or exposure-related screening or surveillance, elevating their need for timely diagnosis. 

Advantages of an RAB program at the VA

Presently, 53 robot-assisted bronchoscopy platforms have been acquired across all VISNs. Establishing a program with positive outcomes is key, and RAB has been shown to yield better results compared to other diagnostic methods [6,7] in detecting lung cancer. Late symptom onset complicates early detection. However, once detected, RAB enables access to small, difficult-to-reach lung nodules, previously requiring more invasive procedures [4]. This in turn facilitates decreased time between diagnosis and treatment. Combining each RAB with mediastinal cancer staging further ensures timely care [13]. Moreover, RAB prioritizes patient safety and comfort by minimizing risks and enabling less invasive procedures, leading to quicker recovery and higher satisfaction [14].

VA pulmonology centers, often linked with top-tier academic institutions, benefit from partnerships that facilitate adopting RAB as standard practice. These collaborations enable the integration of advanced techniques and expertise from academic faculty. While our RAB program predates our academic affiliation’s RAB program, other VA medical systems can leverage this model. Furthermore, the VA plays a crucial role in training fellows and has the potential to become a key platform for educating future healthcare professionals in RAB techniques and improving outcomes for lung cancer patients.

From a financial standpoint, comprehensive cancer care within the VA system reduces instances of veterans seeking care through community care, thus avoiding additional expenses like copayments, deductibles, and transportation costs. Our VA hospital pays an average of $14,867.94 per standard episode of care referral for RAB/EBUS per patient. In-house VA services are tailored to veterans' health needs, with providers attuned to their military service histories, offering specialized support programs, and ensuring continuity of care to enhance patient outcomes.

Challenges

The VA Acquisition Regulation Security Checklist, developed by the Office of Information Technology, serves as a framework for navigating the landscape of security concerns at Malcom Randall VAMC, especially when dealing with equipment that possesses the capability to connect to the VA network or access sensitive privacy information, regardless of whether we intend to utilize these features. Conducting an enterprise risk analysis becomes imperative in such scenarios, a process that demands considerable time and resources. Furthermore, the statement of need highlights the necessity for robust security measures. Documentation from community care paperwork serves to support this need, highlighting the importance of decreasing wait times and demonstrating cost savings by minimizing referrals to external community services.

Engaging with vendors to acquire essential equipment, such as the Intuitive robotic system, often encountered delays in receiving timely responses. While most vendors were very responsive, the overall challenge of coordinating with multiple suppliers impacted the efficiency of the implementation process. We recommend ordering above-par amounts to reduce these wait times and delays. 

Efforts were needed to bridge the disparity between the current and eligible screening population, which has been projected at 30,000 veterans within the NF/SG VHA network according to health factors extracted from CPRS. Continued attentiveness and targeted outreach initiatives are needed to ensure equitable access to lung cancer screening services among eligible veterans [15]. Furthermore, as we increased screening and improved the diagnostic yield of peripheral bronchoscopy, the demand for our RAB services from our network and other networks within our VISN grew. Navigating the logistical aspects of determining the necessary equipment, quantities required, and acquisition timelines posed significant challenges. To cope with the demands to perform more procedures, we started to allocate high-complexity and low-complexity designations to allow us to maximize our procedural schedule.

Efficiently allocating block time with anesthesia and the medical day stay unit (MDSU) nursing team was an additional challenge. To streamline scheduling, optimize resource allocation, and enhance patient care, we implemented guidelines and complexity expectations to minimize disruptions. Additionally, collaborating with the anesthesia team on mitigating modifiable factors for the development of atelectasis with protocolized ventilation and oxygenation strategies has been an important step in reducing RAB procedure time. 

The reprocessing requirements of the Intuitive Ion robotic system presented an opportunity for the SPD to enhance its equipment and expertise. Although temporary reliance on traveling technicians initially posed challenges in mastering the specialized reprocessing protocols, quarterly retraining sessions significantly reduced reprocessing delays.

Future directions

Our enhanced capability to screen and diagnose patients at a higher rate is a testament to our success, though it has stretched our program, resulting in longer waiting periods for nodule sampling. To address demand, our short-term goal is to prioritize the expedited training of additional in-house proceduralists to perform the RAB/EBUS procedure. By expanding our team with skilled professionals, we aim to significantly reduce wait times and enable us to serve more patients efficiently. Furthermore, the long-term objective of our RAB program is to establish our institution as a Referral Center of Excellence for the National VA in the domain of RAB and endobronchial ultrasound (EBUS). We aim to provide exemplary care and expertise to veteran patients across the nation, when not available locally.

In early 2024, we launched a state-of-the-art simulation center for comprehensive RAB and EBUS training, designed specifically for pulmonary and critical care fellows. Looking forward, our goal is to extend training opportunities to VA RAB teams nationwide. This initiative aims to enhance proficiency and competency and promote optimized practices throughout the VA community, fostering collaboration and innovation. By sharing our methodologies and best practices, we aim to provide a framework for developing tailored strategies in individual VA settings, ultimately advancing high-quality care delivery to veterans nationwide.

Lastly, various local transbronchial bronchoscopy ablative treatment approaches are currently being studied. We intend to assess the safety profile, acquire the most safe and effective methodologies, and adapt them for use. Subsequently, we plan to conduct additional clinical trials using these within our network.

## Conclusions

Establishing an RAB program at a VA hospital improves lung cancer diagnosis and treatment among veterans, as late symptom onset and early detection challenges otherwise make lung cancer the leading cause of cancer-related deaths. Implementing RAB at the VA enhances diagnostic precision, streamlines workflow, and facilitates timely interventions, improving patient outcomes. Despite challenges like funding and technological integration, the RAB program at Malcom Randall VAMC shows promise in transforming lung cancer care. This multidisciplinary approach bolsters expertise and comprehensive patient care. Addressing screening disparities and logistical hurdles remains a priority, reflecting the VA's commitment to optimizing healthcare for veterans. Integrating RAB technology into VA systems advances the evidence-based practice of medicine, enhancing veterans' well-being and strengthening the fight against lung cancer.
